# Trends in Iran Pharmaceutical Market

**Published:** 2017

**Authors:** Abdol Majid Cheraghali

**Affiliations:** *School of Pharmacy, Baqiyatallah University of Medical Sciences and Pharmaceutical Management and Economic Research Center, Tehran, IR Iran.*


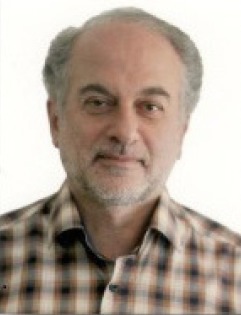


Population of Iran as a Middle Eastern country with its population growth rate of about 1.3% has passed 80 million in 2016. Iran has a young population and it was reported that in 2013 sixty percent of its population was under the age of 30. However, due to a substantial decrease in population growth rate and increase in life expectancy, Iran will face an aging population in coming decades. With an estimated Gross Domestic Product (GDP) in 2015 of 393.7 billion USD Iran is the second largest economy in the Middle East and North Africa (MENA) region (1). Country’s economy depends heavily to oil, agriculture and services sectors and government incomes mainly depend to the oil volatile revenues. A noticeable direct or indirect presence of government in different financial sectors including manufacturing and services is a characteristic of Iran current financial sector. 

Since 1979 Islamic revolution, government of Iran has spent considerable amount of resources on health care services and provided a fairly equitable access for all Iranian to the health care system. As a result in past three decades Iran health sector has achieved considerable success in its goals and this has substantially improved health care system indicators including life expectancy, child-mother mortality rate and access to medicines. Life expectancy of Iranian is substantially above global average and infant mortality have decreased to similar or lower levels to those in countries with comparable income and health spending. In addition to the presence of a country wide primary health care system, secondary and tertiary medical services and high tech medicines are also provided for patients nationwide. However, there are obvious differences between urban and rural access to healthcare services. As a result of interventions, country’s burden of disease has changed from communicable disease to non communicable disease. 

Despite presences both public and private sectors in Iran health care system consists of, Ministry of Health and Medical Education (MOH) is the main policy maker and stewardship of the health care system. According to the country’s constitutional law, Iran government is responsible for providing the highest attainable level of the health care services for Iran citizens. Therefore currently almost all Iranian have access to one sort of state supported health insurance services and national insurance schemes are responsible for reimbursement of medicine costs. These insurance services cover 70% and 90% of the costs of health care services for outpatients and inpatients, respectively. However, due to lack of sufficient resources in public health sectors and national health insurance schemes, in past years and up to 2012 out of pocket payments (OOP) of patients have substantially increased. The share of OOP in total healthcare expenditure was 52.1% in 2013, well above the 15-20% level which has been suggested as the level above which patients financial protection starts to deteriorate in many countries. Since early 2013 in an attempt to reduce OOP paid by the patients, government of Iran implemented a national health reform program. According to the MOH report, following this reform a 32.8% reduction in OOP spending for inpatient services was attained. 

***Iran Pharmaceutical Sector***

The modern pharmaceutical industry in Iran started its activity by licensing the products and process from multinational pharmaceutical companies and importing active pharmaceutical ingredients (API) and formulating them locally. The first modern Iranian pharmaceutical company by private sector established about 70 years ago. The Islamic revolution of 1979 drastically has changed the Iran pharmaceutical industry. Self-sufficiency and non-reliance became main goals of the government. This policy caused the departure of multinational pharmaceutical companies and the new Islamic government nationalized almost all pharmaceutical companies. In order to maintain and further develop national pharmaceutical industry the Iran government in 1980s substantial subsidized hard currency to purchase API and machineries. However, due to lack of substantial investment in the research and development (R&D) area the national industry remained as a formulation based industry. Noticeably, an eight year Iran-Iraq war in 1980s which was started less than two years post Islamic revolution, exerted new pressures on Iran pharmaceutical market. In an attempt to suppress market size and curb medicine consumption, Iran MOH has adapted a full and compulsory generic medicine policy. Based on this policy pharmaceutical companies which were already taken over by the government ask to produce only generic medicines using International Nonproperty Names (INN). Although for more than one decade this policy enforced, following a substantial shift in national policies, Iran MOH has eased its compulsory generic medicine policy. Since 2001 in order to promote competition MOH encouraged national pharmaceutical industry to manufacture branded generic medicines. MOH expected that this policy through creation a more competitive environment eventually improves quality of locally produced medicines. Since then Iran pharmaceutical market witnessed drastic change both in its size and diversity.

In 1990s and following termination of the Iran-Iraq war, government of Iran initiated a nationwide privatization plan for local industry including Pharma industry. In this initiative government transferred ownership of pharmaceutical companies to quasi-governmental organization such as Iran National Social Security Organization (NSSO). In light of new changes in political and economical environment of Iran, MOH revised the National Drug Policy (NDP) on 2004. Regulation of drug market (production, importation and distribution), generic-based medicine policy, promotion of local production of medicines, price control and formulation-based national industry were the main components of the Iran NDP. Iran MOH has insisted on its policy of promoting local production of medicines aims to improve availability and affordability of medicines. Although it is reported that this policy has improved access to the medicines in Iran (2) main challenge of this policy is to define circumstance for successful implementation of the policy. 

It seems that three decades post revolution, young pharmacists who assumed responsibility to manage national pharmaceutical system in 1980s, have reached their maturity and modified their policies. Therefore they started a national plan for establishment of a more relax and market based pharmaceutical market. However, despite implementation of national scheme for privatization of the industry still majority of the local pharmaceutical market is in hand of semi governmental organizations. It is reported that Tamin Investment Corporation (affiliated to NSSO), Barkat Pharmaceutical Corporation (affiliated to office of supreme leader of Iran) and Shafadaru Corporation (affiliated to Bank of Melli, a state owned bank) hold more than 50% of the Iran local pharmaceutical market (3) Therefore, despite the fact that private pharmaceutical companies gained more market share in Iran in recent years, their share in Iran pharmaceutical market is still below 50%.

All aspects of medicines policy including production, importation and distributions of medicines in Iran are under strict control of Iran Food and Drug Administration (IFDA). The first parliamentary law governing food and medicines in Iran was passed in 1955. This law with some modification is still in effect (4). Therefore all medicines should receive registration and marketing authorization from IFDA before entering the Iran market. In Iran medicine prices are also under regulation of the IFDA. Despite liberalization in governmental regulations and reduction of red tapes in recent years there is still substantial influence from governmental decisions on the sector.

In related to its nuclear activities, in past decade Iran faced diverse regional and international sanctions and restrictions on different fields including gas and oil, banking and insurance sectors. As a result of these sanctions and restrictions Iran industrial sector including local pharmaceutical industry faced profound difficulties for procurement of machinery, technology and even finished products and API. This happened mainly due to difficulties to access lines of international credits for Iran pharmaceutical companies. In the other hand and due to reduction of the country’s international incomes, Iran national currency (Rial) lost its value against international currencies. These have caused both substantial price increase and shortage of the medicines in Iran pharmaceutical market (5). Signing of the Joint Comprehensive Plan of Action between Iran and the P5+1 (i.e., China, France, Germany, Russia, the United Kingdom and the United States) in November 2013 which followed by lifting or easing of the nuclear activity related restrictions provided an improvement for Iran’s economy and the related rise in business confidence. Meanwhile Iranian policy makers have adopted a comprehensive national plan for boosting market-based economy for the country. This policy reflected in the government’s 20-year vision document and the sixth five-year development plan for the 2016-2021 period. Therefore it is expected that in coming years Iran economy including pharmaceutical market will be expanded and private sector will gain more share in Iran pharmaceutical market.

***Current Pharmaceutical Market Situation***

As a result of demanding high tech and expensive biopharmaceutical medicines both by prescribers and patients, healthcare expenditures in Iran have risen in recent years. Recent published trend analysis of Iran pharmaceutical market shows that the market has been drastically grown in the recent decade. In the 1997-2010 period, despite a total and annual growth of 21.3% and 1.53% in the total country’s population and population growth rate, over the 13-year period, the data indicates that the per capita expenditure of medicines has reached 34.43 USD from 2.28 USD in 1997 and an annual average growth of 10.8% (3) 

As depicted in Figure 1 in 2015 Iran pharmaceutical market valued over 4.3 billion USD (based on official exchange rate for national currency and USD(. Per capita expenditure on medicine in Iran in 2015 stood at about 54 USD. Due to fairly advanced health care system and presence of substantial numbers of specialty physicians for providing secondary and tertiary medical interventions, Iran pharmaceutical market has the good potential for expansion in light of post sanctions era. This expansion would most likely happen in high tech medicines including monoclonal antibodies (mAbs) and recombinant proteins. 

 Compared to many other countries of the region Iran pharmaceutical market could be considered as a large and lucrative market. Iran pharmaceutical market has experienced an almost 6 times increase in market value between 2008 to 2015 (Figure1). However, it should be mentioned that most of this increase is due to national currency (Rial) devaluation. Rial has experienced several drastic devaluation in past three decades. For example official exchange rate for Rials to USD which was 9,100 Rials for one USD in 2004 has increased to 31,500 Rials in 2015. This obviously influenced pharmaceutical market which heavily depends on international currencies. Therefore most part of increase in pharmaceutical market as shown in Figure 1. is actually due to devaluation of national currency. However, there is still an about 15% gap between official exchange rate for national currency with that of free market exchange rate. It seems that government of Iran plans to remove this gap in near future through liberization of hard currency exchange rate. Therefore it is expected that following floating the local currency and subsequent currency depreciation, pharmaceutical market might experience an increase in value in 2017.

Traditionally Iran Drug Selection Committee, responsible body for developing and revising Iran Medicine List, used to request acceptable safety, quality and efficacy data for including new medicines to the List. Recently, in shadow of pressures from limited available resources in national health care system, Iran policy makers use pharmacoeconomics considerations to evaluate benefit of medicines in comparison with their extra costs to the health care system (6). Therefore, it is expected that downward pressure on medicines prices remains in place for coming years as the purchasing power of Iranian patients is lower than that of some other countries of the region.

Despite the fact that promoting of national pharmaceutical industry is one of the main goals of the Iran NDP, share of national pharmaceutical industry in local pharmaceutical market is still about 60% (Figure 2). Iran has one of the largest capacity of production of generic medicines in the Middle East and MENA region. While per capita consumption of generic medicines is acceptable, access to specialized medicines for treatment of life threatening medicines is restricted due to limited resources available in health care system. Therefore, most imported medicines are including biotherapeutics, recombinants and oncology medicines.

Imposing high tariffs on imported medicines is one of the major policies that the government of Iran implemented to support the local pharmaceutical industry. In another development MOH encourages the local industry to manufacture copied s locally. MOH believes that this policy will benefit patients through improvement of availability of the locally manufactured biopharmaceutical for effective treatment of chronic and life threatening diseases. Few newly established pharmaceutical companies in Iran are involved in manufacturing of similar biopharmaceuticals for local market. Copied biopharmaceuticals produced by local companies received marketing authorization for the local market based on national guidelines for evaluation of these products. Among the biopharmaceuticals produced in Iran over the past decade are inteferons, growth hormone and erythropoietin and several mAbs (7).

Local pharmaceutical sector in Iran suffer from some drawbacks. It suffers mainly from poor GMP structure in some parts of local industry. The average age of pharmaceutical companies in Iran is over half century old and desperately needs renovation in its facilities and equipments. Lack of new investments on large scale old companies is an important hurdle for upgrading the facilities. Their needed investments are substantial and their major shareholders which are mostly semi-governmental organization are not interested to do such investments. However, it seems that any renovation activities of local manufacturing facilities will accompany with improving GMP standards and this will boost potential for export especially to the countries of the region. In presence of a fairly large local market, pharmaceutical companies in Iran have a good opportunity for exporting their products to some neighboring countries including Iraq, Afghanistan and CIS countries. Lack of effective R&D activities and marketing strategies are also main weakness of Iran pharmaceutical market. Marketing strategies of local pharmaceutical companies are poorly developed and mostly focused on price war by providing discount offers to the pharmacy outlets. Companies encourage pharmacies to buy more and pay less such as buy three and pay for two. 

**Figure 1:**
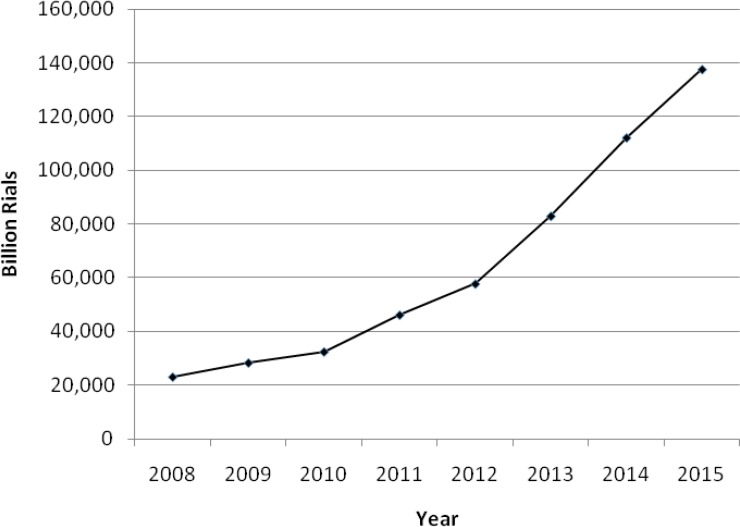
Trend of Iran Pharmaceutical market 2008- 2015 (official exchange rate in 2015; 1USD = 31,500 Rials).

**Figure 2:**
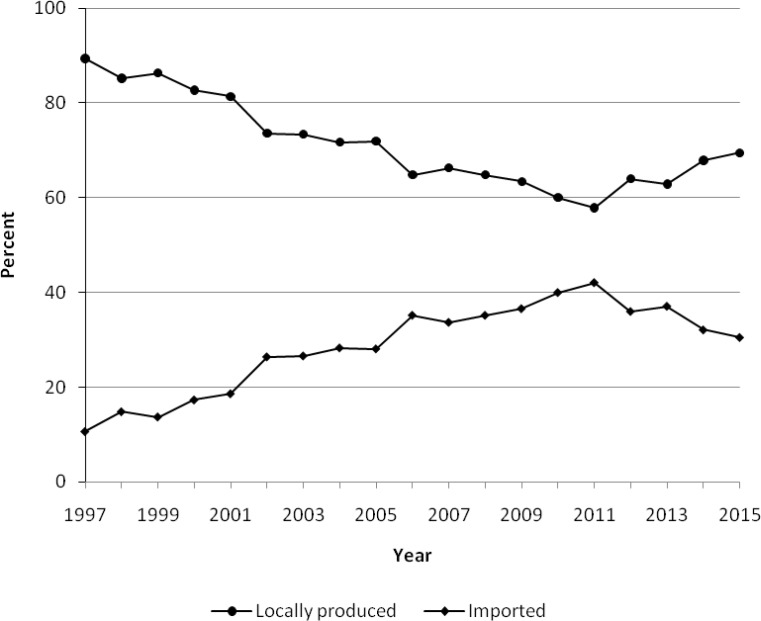
Share of locally manucatured and imported medcines in Iran pharmaceutical market 1997-2915.

Due to lack of efficient R&D projects in local pharmaceutical companies it is unlikely to produce new break through products for years to come. Therefore, the overall technological capacity of national pharmaceutical industry still limited to manufacturing and formulation of generic medicines. However, in a new development in Iran’s pharmaceutical sector some private knowledge based local companies have started to produce biopharmaceuticals and high tech medicines including copied high tech biological medicines using novel biotechnology methods (7). 

In another new development Iran MOH announced its support for international investment into Iran pharmaceutical market. Iran plans to provide acceptable environment in order to attract foreign investment for pharmaceutical industry in order to upgrade its technology capabilities and manufacture high tech medicines such as mAbs and recombinant proteins. Key components such as expertise, skilled labor, low cost of production, geopolitical location, and infrastructure and support of national authorities have made Iran a potential export hub in the region. Therefore, some well known international pharmaceutical companies such as Novo Nordisk, Sanofi and Novartis have announced their intentions and plans for investment in Iran pharmaceutical market.

Lack of intellectual property rights (IPR) protection law is also a concern for international Pharma companies. So far international pharmaceutical companies mostly operate in Iran through providing dealership to Iranian companies to sell their products. Interestingly some Iranian companies represent more than one foreign company and sometimes rival companies. However, recently IFDA encourages international companies to establish their direct operating office in Iran and mange all aspects of their medicines in Iran market through their own office. Despite presence of laws for protecting patents and IPR in Iran Since 1925, still there is no comprehensive IPR law to provide full protection of innovations as general and especially in the pharmaceutical sector. Therefore currently IFDA licenses medicines from different producers and importers. Iran intended to become full member of WTO and this obviously will have drastic impacts on both national pharmaceutical industry and pharmaceutical market in Iran. On that time, due to restrictions on the presence of copied patent medicines on the market, the government of Iran has to allocate substantially more resources for providing high tech medicines to the national health care system.

Although pharmaceutical companies can market or manufacture a product after obtaining a license from the MOH, such licensing is given to more than one company in any case. At present, there is no developed patent protection, especially for imported medicines in Iran IFDA registers copied products of patent medicines. 

## Conclusion

Due to recent developments in economical environment in Iran, it is expected that Iran pharmaceutical sector will expand both in value and volume. In recent decade some private companies also emerged in Iran pharmaceutical market. Emerging of newly established small knowledge based companies might pose both risks and opportunities for Iran pharmaceutical market. These new companies have fragile financial turn over and recent government policies of subsidy reforms which resulted to the hike in energy costs and hard currency floating policy might put some pressure on local manufacturing in general including the new high tech companies. 

Despite presence of local pharmaceutical companies, due to fairly advanced health care system of Iran, the country needs to import expensive novel medicines to satisfy patients’ demands. Iran pharmaceutical market is considered as a highly regulated market. As related to the national pharmaceutical market generally the IFDA makes all strategic decisions and monitor implementation of the regulations. 

Although in an attempt to improve transparency of procedures, in recent years IFDA has published its policies and guidelines, lack of procedural transparency, lack of consistency and non binding to the regulations are major weaknesses of IFDA. In its current administration system in IFDA offices, different pharmaceutical companies might receive different responses for similar requests. Therefore in some cases IFDA may decide based on “case by case” approach. Lack of efficient control on promotional activities of pharmaceutical companies is also a derive for inducing demands in Iran market. Despite presence of a governing guideline in IFDA for regulation of promotional activities of pharmaceutical companies, relax implementation of the guideline provided an almost absolute liberty for companies on their relations and sometimes misconduct with prescribers and physicians in order to bring their brands into their prescriptions. There is a danger that lack of transparency and uncertainty in the regulatory requirements for the registration of the medicines will encourage manufacturers to ignore ethical aspects of promotion. This may cause irrational use of medicines which could impose extra expenses both in financial and medical aspects on patients and the national health system. 

## Competing interests:

**None**

## Note

*The author is a member of GaBi international advisory board.
